# Transcriptome-wide analysis of immune-responsive microRNAs against poly (I:C) challenge in *Branchiostoma belcheri* by deep sequencing and bioinformatics

**DOI:** 10.18632/oncotarget.20570

**Published:** 2017-08-28

**Authors:** Qi-Lin Zhang, Qian-Hua Zhu, Feng Zhang, Bin Xu, Xiu-Qiang Wang, Jun-Yuan Chen

**Affiliations:** ^1^ State Key Laboratory of Pharmaceutical Biotechnology, School of Life Science, Nanjing University, Nanjing, China; ^2^ LPS, Nanjing Institute of Geology and Paleontology, Nanjing, China; ^3^ Beijing Genomics Institute, Shenzhen, China

**Keywords:** Branchiostoma belcheri, miRNA, poly (I:C), deep sequencing, Immunology and Microbiology Section, Immune response, Immunity

## Abstract

Amphioxus is a key experimental animal for studying the evolution of vertebrate immune system. However, we still do not know about the roles of microRNAs (miRNAs) under viral stress in amphioxus. In this study, we sequenced six small RNA libraries (three biological replicates were included in the treatments challenged by the viral mimic, poly (I:C) (pIC) and control groups, respectively) from *Branchiostoma belcheri*. A total of 151 known miRNAs, 197 new miRNAs (named novel_mir, including nine conserved miRNAs) were identified by deep sequencing from the six libraries. We primarily focused on differentially expressed miRNAs (DEMs) after pIC challenge. Next, we screened a total of 77 DEMs, including 27 down- and 50 up-regulated DEMs in response to pIC challenge. Furthermore, we used real-time quantitative PCR (qRT-PCR) to verify the expression levels of 10 randomly selected DEMs. Target genes likely regulated by DEMs were predicted, and functional enrichment analyses of these targets were performed using bioinformatics approach. MiRNA targets of DEMs are primarily involved in immune response, diseases, cancer and regulation process, and could be largely linked to 14 immune-related signaling pathways, including NF-kappa B, NOD-like receptor, RIG-I-like receptor and endocytosis. The present study for the first time explores key regulatory roles of miRNAs in the innate antiviral immune response in amphioxus, and will provide insight into the molecular basis of antiviral immunity and evolution of immune-related miRNAs.

## INTRODUCTION

Sister subphyla, the Cephalochordata, Urochorda, and Vertebrata, is all the three members of phylum Chordata [1]. Amphioxus is the living representative of Cephalochordata, and is considered to be the last common ancestor of chordates owing to its proximal morphological and genomic evidence with the ancient chordate group [2, 3]. This group usually burrows shallow in temperate and tropical seas with growth fuel of microorganisms [4]. Amphioxus must thus face acute invading microorganisms, which facilitates adaptive evolution of its innate immune system [4]. Indeed, many experiments reported that amphioxus is susceptible to certain bacteria, bacterial and viral mimics, such as lipopolysaccharide (LPS) and poly (I:C) (pIC; a double-stranded RNA viral mimic) [5-7]. Many studies have found acute changes in expression of genes/microRNAs (miRNAs) upon immune challenge in amphioxus [8-10]. Thus, amphioxus can serve as a model for investigating primitive innate immune responses, signaling pathways and exploring the evolution of key immune-related molecules.

miRNAs, a class of endogenous non-coding small RNAs, are transcribed initially by RNA polymerase II, which are then processed by Drosha and Dicer-1 enzymes to form miRNA: star-miRNA duplexes[11]. Following cleavage by Dicer, more stable miRNAs (approximately 22 nt) preferentially incorporate into an RNA-induced silencing complex (RISC). Next, miRNAs guide the RISC to target complementary sequences in the 3’ UTR (untranslated regions) of mRNAs to negatively regulate gene expression, thereby blocking the translation of target transcripts or degrading mRNA [12-14]. Many previous studies have recognized that miRNA molecules are important regulators of immune, inflammatory and disease response in animals [15-17]. For example, some miRNAs, such as miR-155, miR-223, miR-181a, miR-17-92 cluster and miR-146 family mediate expression of target genes associated with immune cell development and function in mammal hematopoiesis [18]. Previous studies speculated that miR-146a, miR-155 and miR-9 suppressed the acute responses following activation of innate immune system by negatively regulating the expression of genes involving receptor-induced signaling pathways in vertebrates [19]. Lu et al. found that the absolute decrease in abundance of many microRNAs, including the *let-7* family, could significantly promote oncogenes is in human cancer [20]. Recently, Jin et al. identified 14 differentially expressed miRNAs (DEMs) in *Branchiostoma belcheri* infected with *Vibrio parahemolyticus* when compared with the control using microarray profiling [10]. Liao et al. detected 56 DEMs in gills of *B.belcheri* after LPS challenge using microarray technology [9]. Yang et al. found that bbe-miR-7, bbe-miR-4868a, bbe-miR-2065, bbe-miR-34b and miR-92d were differently expressed in *B. belcheri* upon infection with both *Vibrio anguillarum* and *Staphylococcus aureus* [21]. These studies focus on roles of miRNAs in immune response of amphioxus only to challenges with bacteria or bacterial mimics. However, no studies have been done yet to analyze the function of miRNAs in the antiviral immune defense in amphioxus.

Currently, with the development of high-throughput miRNA deep sequencing technologies, whole-transcriptome/genome identification and expression analysis of miRNAs has been widely conducted to massively and efficiently screen for DEMs involving immune responses and explore their roles in vertebrate immunity [22, 23]. Additionally, in previous immune-related studies in amphioxus, hybridization-based microarray analysis has been the predominant method for detecting acute immune responses of miRNAs to challenges with pathogens and their mimics [9, 10]. However, recently, Jin et al. systematically identified expression of miRNAs under bacterial infection using miRNA Solexa deep sequencing, thus providing a full suite of candidate probes for subsequent preparation of microarrays to analyze differential expression of miRNAs [10]. Evidently, when compared with hybridization-based technologies such as microarrays, miRNA deep sequencing can identify miRNAs expressed even at low abundance and reliably read sequences of miRNAs among different samples [24]. Thus, deep sequencing has been a powerful approach for analysis of transcriptome-wide immune-responsive miRNAs in amphioxus species with sequenced genomes, including *Branchiostoma floridae* and *B. belcheri* [2, 25].

In the present study, to investigate the miRNA expression changes in antiviral immunity and to provide insights into the regulated roles of miRNA in immune signaling pathways in amphioxus, we performed transcriptome-wide analysis of immune-responsive microRNAs under pIC challenge in *B.belcheri* using deep sequencing and bioinformatics methods. Known, conserved and novel DEMs were identified using the bioinformatics approach. We then performed quantitative real-time PCR (qRT-PCR) analysis for a subset of randomly selected DEMs to validate the sequencing data. To further understand the functions of these DEMs, the corresponding target genes were predicted and then analyzed using enriched Gene Ontology (GO) terms and Kyoto Encyclopedia of Genes and Genomes (KEGG) pathways. These findings should be helpful for better understanding the regulatory roles of miRNAs in antiviral immune response of *B. belcheri*.

## RESULTS

### Analysis of small non-coding RNAs

**Figure 1:**
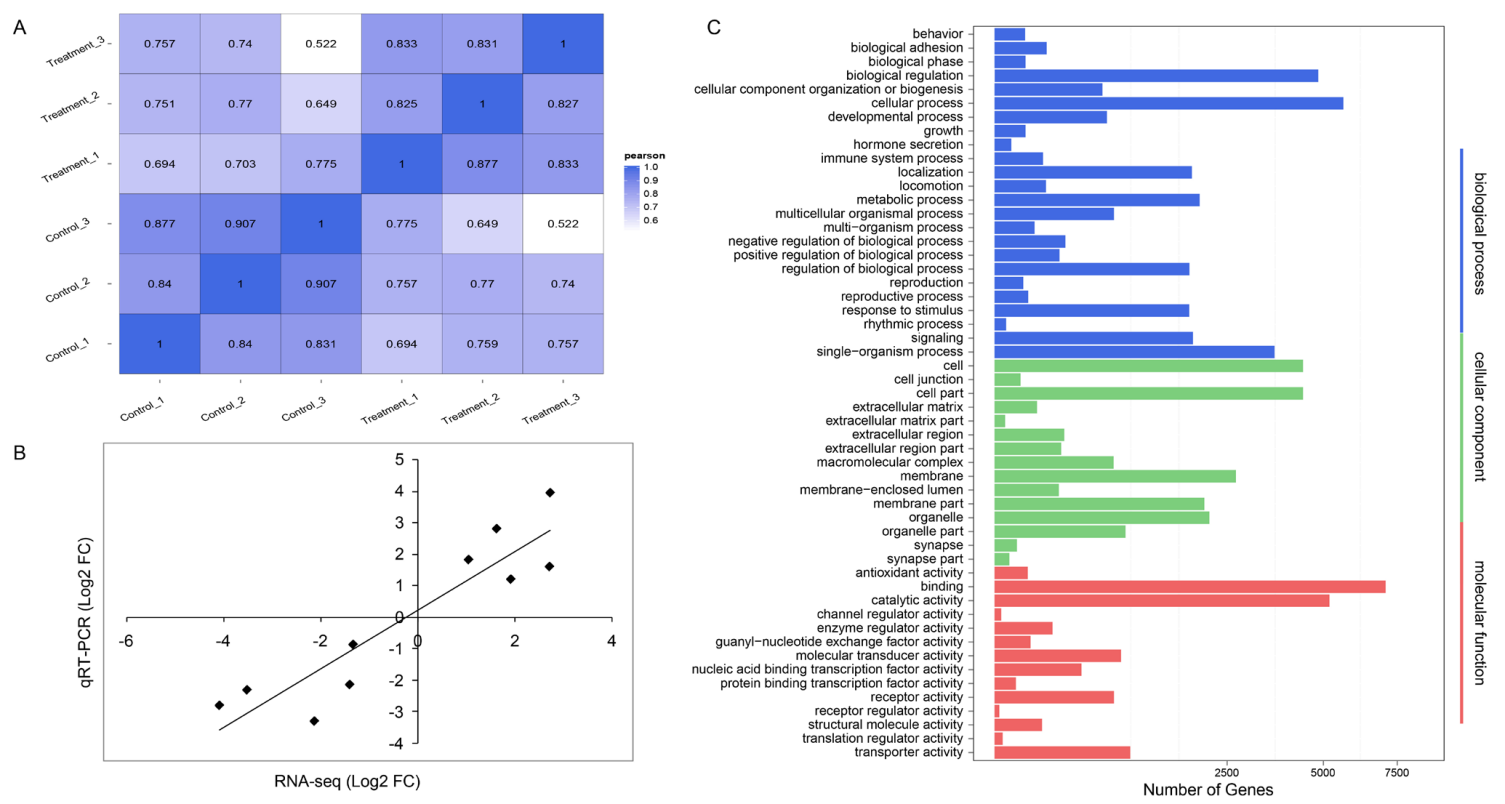
Reliability of deep-sequencing data and functional enrichment analysis **A.** Heatmap of Pearson correlation coefficient among different samples. The maximum and minimum values are marked by blue and white respectively. A higher value indicates that the two samples are more similar. **B.** Correlation of Log2 (fold changes) in miRNA expression between deep sequencing and qRT-PCR results. The relative expression fold change between the control and treatment groups is indicated by black dots. **C.** GO terms enriched among target genes of differently expressed miRNAs. The x-axis indicates the number of target genes, and the y-axis shows the GO terms. All terms are classified into three subcategories marked by different colors.

To identify immune-responsive miRNAs upon pIC challenge in *B.belcheri*, six small non-coding RNAs (sRNAs) libraries were sequenced from treated or control whole bodies of adult *B.belcheri* (three biological replicates for each of the treatment and control), generating 28.9, 29.6, 30.1 million (control groups) and 31.3, 29.9, 30.1 million (treated groups) raw tags. After performing quality control, 28.2, 28.7, 29.2 million clean tags were obtained from control samples, and corresponded to 97.63%, 97.13% and 97.01% of the total raw tags respectively; we obtained 30.0, 28.8, 29.2 million clean tags from treated samples, constituting 95.74%, 96.51% and 97.05% of the total raw tags respectively. The ratios between total mapped tags to the *B.belcheri* genome and clean tags ranged from 78.82% to 83.74% (Supplementary Table 1). Based on the transcripts per million (TPM) values of all detected sRNAs, Pearson’s correlation values were calculated for pairwise comparisons among the six libraries. The values were > 0.80 for comparisons among the three biological replicates from similar treatments, i.e., among the negative controls or among treated groups (Figure 1A), indicating that the experimental approaches in this study were reliable.

The length distribution of clean tags in the six libraries was calculated, and sizes of sRNA were found to range from 17 to 33nt (Supplementary Figure 1). In all libraries, the most abundant length of sRNA was 22 nt, followed by 23 nt and 21 nt, which is consistent with the normal length of miRNAs in *B.belcheri* [10] and other animals, including *Larimichthys crocea* [22] and *Bombyx mori* [24]. The results showed that the percentage of miRNA tags increased from ∼30% in negative control groups to ∼40% in pIC-treated groups (Supplementary Table 1), indicating that miRNA molecules play a key regulatory role in antiviral immune response of *B.belcheri* to pIC challenge. We detected 162, 165 and 164 known miRNAs in the three control group replicates, of which 132 known miRNAs were shared among all three libraries, and were considered to be the known miRNA set of negative control for further analysis. Similarly, 169, 168 and 168 known miRNAs were found in the treated groups, of which 136 were common and considered as known miRNAs. By getting union of known miRNAs between the control and treated groups, we obtained a total of 151 known miRNAs for downstream screening of DEMs. Based on the total expression level (average TPM value of treatment plus that of control), we found that *let-7*, mir-10, mir-100 were the top three among highly expressed miRNA families, while members of the mir-10 family were the most abundant in the list of top 30 miRNAs (Supplementary Table 2).

### Identification of conserved and novel miRNAs in *B. belcheri*

We found 211, 222 and 215 novel miRNA sequences in three biological replicates of control groups, while 217, 220 and 214 novel miRNA sequences were obtained in the treated groups (Supplementary Table 1). By comparing the biological replicates, a total of 147 and 152 novel miRNA were shared within the control and treated groups respectively. Finally, we identified a total of 197 novel miRNA sequences by getting union for control and treated groups, which were analyzed further for DEMs. Novel-mir51, novel-mir2, novel-mir108 were the top three novel miRNAs with high expression levels (Supplementary Table 3). Specifically, novel-mir51 showed the most abundant expression, which far exceeded that of other novel miRNAs, and was predicted to be a regulatory molecule of target genes encoding ribosomal protein S10 (S10). Furthermore, to investigate the conservation of these 197 novel miRNAs among animals, we identified nine conserved miRNAs belonging to eight families (Table 1). The homologs of the other novel miRNAs were not found, suggesting that they were not conserved in other animals and may potentially be *B. belcheri*-specific. Moreover, among the conserved miRNAs, sequences of five miRNAs (novel_mir3, novel_mir14, novel_mir24, novel_mir65, novel_mir108) have been reported to be involved in the immune response of *B. belcheri* to *V. parahemolyticus* infection [10]. Except for novel_mir152, eight out of a total of nine conserved miRNAs exist only in *B. belcheri* and *B. floridae*, indicating that they are novel amphioxus-specific miRNAs.

**Table 1:** Nine potential conserved *Branchiostoma belcheri* miRNAs newly identified by deep RNA-seq.

Mature sequences (5’- 3’)	miRNA id	Homologous miRNAs
acgcguuuaugucugcgccugu	novel_mir2	bfl-miR-4862-5p
ucgcauugacgucagcgccggc	novel_mir3	bfl-miR-4856b-5p
uagcuggcuggcgaagaaaug	novel_mir14	bfl-miR-4900a
uagcuggcuggcgaagaaaagc	novel_mir24	bfl-miR-4900a
aucuggggcaauuaagguuacga	novel_mir26	bfl-miR-4894
uuuggcacugguacuuuggagu	novel_mir65	bfl-miR-4871-3p
gauguuuguacugucugucuguu	novel_mir108	bfl-miR-4870
caagcucgugucuaugguucu	novel_mir136	bfl-miR-100-3p
ugagguaguagguuguagg	novel_mir152	hsa-let-7c-5p, mmu-let-7c-5p, rno-let-7c-5p, gga-let-7c-5p, dre-let-7c-5p, ssc-let-7c, xtr-let-7c, etc

Bfl indicates Branchiostoma floridae; hsa, Homo sapiens; mmu, Mus musculus; rno, Rattus norvegicus; gga, Gallus gallus; dre, Danio rerio; ssc, Sus scrofa; xtr, Xenopus tropicalis.

### Identification and qRT-PCR validation of immune-responsive miRNAs

We obtained 151 known and 197 novel (including nine conserved) miRNAs that were detected in at least one of the control and experimental groups. These were considered as candidates for further differential expression analysis. A total of 77 miRNAs, including 33 known and 44 novel (including two conserved) miRNAs, were identified to be differentially expressed in immune response to pIC challenge, of which 50 and 27 miRNAs were detected to be up- and down-regulated, respectively (Table 2). Among the DEMs, novel-mir27 was the most up-regulated, followed by novel-mir183 and novel-mir121. These three novel miRNAs were predicted to regulate target genes encoding lipoxygenase 2 (LOX2), retinoic acid receptor (RAR), and putative oxidoreductase GLYR1 respectively by bioinformatics. Conversely, the top three DEMs that were most down-regulated after pIC challenge were novel-mir138, novel-mir158 and novel-mir140. These were predicted to regulate genes encoding heat shock 70 kD protein (HSP70), nucleotide-binding domain, leucine rich containing (NLR) protein family, and complement C1q-like protein (C1qL) respectively. Among known DEMs, the greatest up-regulation in expression was observed for bbe-miR-31-3p and bbe-miR-4856a-5p, which exhibited more than 3-fold increase in abundance when compared with the control groups. Conversely, bbe-miR-2070-5p and bbe-miR-252a-5p were the most down-regulated (more than 4-fold changes) miRNAs after pIC challenge. The fold changes of 10 randomly selected DEMs between the deep sequencing and the qRT-PCR data were found to be significantly positively correlated (Pearson’s correlation coefficient = 0.821, two-side *P-*value < 0.01) based on the linear regression analysis method (Figure 1B), indicating the accuracy of the miRNA deep sequencing method.

**Table 2:** Differentially expressed miRNAs (DEMs) in the whole body of *B. belcheri* challenged with pIC when compared with the negative control, as identified by deep sequencing. # indicates conversed DEMs.

Mature sequences (5’- 3’)	miRNA id	log2 (fold change)	Regulation pattern	FDR
uucgaaaucucucuguuccau	novel_mir27	8.40	UP	3.02E-97
uaaccuguaaaucggauuugu	novel_mir183	8.10	UP	3.54E-82
uguuaaauuaaugcuaugccac	novel_mir121	7.52	UP	1.01E-58
uccuggguuggcuguuggcggcac	novel_mir196	7.25	UP	3.10E-50
ucugggugucauccuccguagu	novel_mir33	6.89	UP	1.56E-40
accccgacccggccuggcg	novel_mir95	6.04	UP	1.64E-24
ucgcguaccgucugguuguggu	novel_mir59	5.83	UP	1.57E-21
ucuucuauggcccgggaucuu	novel_mir35	5.56	UP	2.39E-18
uuaggacauguucuacagcu	novel_mir157	5.56	UP	2.39E-18
aacugggaccaugucuuccugc	novel_mir85	5.28	UP	1.51E-15
aucauacaaaaggauuaccgagu	novel_mir116	5.09	UP	6.97E-14
uuuagugaacuaugcaccaagu	novel_mir90	4.89	UP	2.38E-12
ucagagaagccgagcugucaga	novel_mir156	4.23	UP	1.91E-08
ucccccaguccggcggcaacgu	#novel_mir136	4.13	UP	5.73E-08
uuggccggccauggagggucc	novel_mir115	4.05	UP	1.17E-07
ucgauggccggcugccuucucu	novel_mir81	3.81	UP	1.07E-06
cgagucgucugauugcguuugc	novel_mir49	3.76	UP	1.54E-06
gctgtgctgacaaactgccgtt	bbe-miR-31-3p	3.66	UP	0.00E+00
acgcagtgacgtcagcgcctct	bbe-miR-4856a-5p	3.48	UP	0.00E+00
agcgccgacggugguacacucucc	novel_mir38	3.39	UP	2.11E-05
uuuggcacugguacuuuggagu	#novel_mir65	3.20	UP	6.28E-05
acuccucuggcauguuauuugu	novel_mir48	3.20	UP	6.28E-05
aguaccgccagagagaccuuu	novel_mir147	3.16	UP	0.00E+00
ucggagugagauuucaggguggcgg	novel_mir160	3.13	UP	9.07E-05
caatgtaacagcagtgcagct	bbe-miR-33-2-3p	2.76	UP	0.00E+00
cccttatcacttcttccgccgag	bbe-miR-184-5p	2.73	UP	1.27E-97
aauccacagugauuuuggccaugu	novel_mir193	2.71	UP	5.64E-04
aacaactaacatcactgccaag	bbe-miR-34b-3p	2.71	UP	0.00E+00
aggaagacauggucccaguugu	novel_mir50	2.67	UP	0.00E+00
uuaugauuaucgagagaguucguu	novel_mir99	2.61	UP	8.03E-04
taccctgtagatccggacttgt	bbe-miR-10c-5p	2.52	UP	0.00E+00
uaguguugcgacgaagaauagc	novel_mir36	2.43	UP	6.57E-23
cgcctctgtttcgcgcgcaag	bbe-miR-210-3p	2.24	UP	8.59E-76
gtgattagctaaggtagctgata	bbe-miR-4890-5p	2.20	UP	1.01E-05
cttaccaggcagcatttagt	bbe-miR-200b-3p	1.92	UP	0.00E+00
ucuugggcauguagguuaugag	novel_mir150	1.79	UP	7.27E-201
agucaccgucugucggcccguacga	novel_mir114	1.63	UP	7.60E-183
uacuggccuccaaagucccggu	novel_mir10	1.58	UP	2.99E-19
accaatatatcgaacatatgat	bbe-miR-190-3p	1.55	UP	0.00E+00
auacggggcaguuauauccaggu	novel_mir170	1.51	UP	2.42E-16
uacggcuccccaagccgugguu	novel_mir31	1.47	UP	3.79E-21
ctgtgcaacctgctagctctcc	bbe-let-7a-2-3p	1.45	UP	0.00E+00
gcagactggtgcttaacat	bbe-miR-252a-3p	1.36	UP	0.00E+00
uggcaguguauuuagcuggguaa	novel_mir52	1.30	UP	8.87E-28
aucuggggcgguuaagguuauga	novel_mir26	1.16	UP	1.63E-35
uggccuaaucccuguggauuu	novel_mir20	1.11	UP	1.58E-32
tgcaacaatatttcatcagtgg	bbe-miR-2062-5p	1.08	UP	0.00E+00
ttctcgattgttctggtcca	bbe-miR-4887-5p	1.05	UP	0.00E+00
auucggguuuauaggaacauga	novel_mir19	1.04	UP	7.93E-49
gactgtacaacccgttacctt	bbe-let-7a-3p	1.01	UP	0.00E+00
acacuuauaggauuucucaccu	novel_mir166	-1.09	DOWN	2.25E-26
cagtaaatcacagtcttcctta	bbe-miR-7-3p	-1.17	DOWN	0.00E+00
tgcagagcttgtgcgattggcg	bbe-miR-4888-5p	-1.23	DOWN	2.63E-35
agacaacatcagaatgtccct	bbe-miR-4863-5p	-1.32	DOWN	1.82E-17
agggacctgtagtcaacacgaca	bbe-miR-4869-3p	-1.33	DOWN	0.00E+00
cacuggcccucuacgucuuga	novel_mir1	-1.34	DOWN	0.00E+00
tattgcacttatcctggcctgt	bbe-miR-92d-3p	-1.40	DOWN	0.00E+00
tgttcccaccttctgatgttgt	bbe-miR-4873-5p	-1.58	DOWN	0.00E+00
tgcgtaggcgttgtgcacactgt	bbe-miR-242-5p	-1.59	DOWN	4.20E-48
aggtctggatagttgcaatctt	bbe-miR-92b-5p	-1.66	DOWN	5.49E-11
gcttagcagtgcaagtgtgac	bbe-miR-4866-3p	-2.04	DOWN	0.00E+00
caucgacgccguccguuguagacgc	novel_mir130	-2.12	DOWN	0.00E+00
caatgtaactgcagtgcagcc	bbe-miR-33-3p	-2.16	DOWN	0.00E+00
atgcggtgcggtggtagcaaccg	bbe-miR-2071-3p	-2.21	DOWN	0.00E+00
gggcatgtgtgcatagggaaga	bbe-miR-2068-3p	-2.51	DOWN	0.00E+00
gttcagcaccaaggacaggc	bbe-miR-2060c-3p	-2.60	DOWN	6.17E-47
actggtgaaatgtagttgcgta	bbe-miR-2062-3p	-2.98	DOWN	0.00E+00
uugggagucaggacaccauuguggu	novel_mir148	-3.51	DOWN	8.24E-180
cttggcacttgtggaattctctg	bbe-miR-182b-5p	-3.65	DOWN	7.92E-09
caucgacgccguccguuguggac	novel_mir131	-3.80	DOWN	1.06E-40
aattgcactagagtgatttgtt	bbe-miR-2076-5p	-3.90	DOWN	0.00E+00
tttccacagcctctacacatgt	bbe-miR-2070-5p	-4.08	DOWN	0.00E+00
ctaagtactagtgccgcaggtgt	bbe-miR-252a-5p	-4.64	DOWN	0.00E+00
ugcucugagaugauaguaccagu	novel_mir194	-6.65	DOWN	6.91E-10
uucagaggacguaaacgcuuug	novel_mir140	-6.89	DOWN	4.39E-11
acuggguccagccggcucucccug	novel_mir158	-7.19	DOWN	8.12E-13
gccgagccggggcaggucgccccc	novel_mir138	-8.30	DOWN	0.00E+00

### Prediction of target genes and functional enrichment of immune-responsive miRNAs

We used two prediction methods to predict the target genes of all DEMs. These putative target genes were then assigned to the three standard subcategories of “Biological Process”, “Cellular Component” and “Molecular Function” in GO enrichment analysis (Figure 1C). In the “Biological Process” subcategory, 24 terms were enriched with the most abundant genes being involved in cellular processes, followed by biological regulation, and single-organism process. Notably, three terms directly related to immune response, including immune system process, response to stimulus and signaling, were also detected. In the “Cellular Component” subcategory, 15 terms were assigned and the most abundant genes were related to cell, cell part and membrane. Lastly, 14 terms were assigned into “Molecular Function”, of which binding and catalytic activity had the most abundant genes.

Furthermore, the target genes of all DEMs were also classified into 42 KEGG pathways (Supplementary Table 4), of which 14 are immune-related pathways listed in Table 3, and include NF-kappa B signaling pathway, NOD-like receptor signaling pathway, MAPK signaling pathway, and RIG-I receptor signaling pathway, and so on. Since the pathogen can directly induce immune response and cause disease in the host, we classified disease-related KEGG terms along with immune-related pathways and found that many of them are involved in important virosis in vertebrates, such as viral carcinogenesis, hepatitis C, Influenza A, etc.

**Table 3:** List of the primarily immune-related KEGG pathways of target genes of differentially expressed miRNA (DEMs) between the *B. belcheri* control and that challenged with pIC

**Pathway**	**Pathway ID**	***P*-values**	**FDR**
NF-kappa B signaling pathway	ko04064	7.70E-05	2.63E-03
NOD-like receptor signaling pathway	ko04621	8.64E-05	2.65E-03
MAPK signaling pathway	ko04010	1.10E-04	2.90E-03
Endocytosis	ko04144	1.38E-04	3.03E-03
Chemokine signaling pathway	ko04062	1.97E-04	3.78E-03
Viral carcinogenesis	ko05203	2.11E-04	3.81E-03
Salmonella infection	ko05132	3.77E-04	6.09E-03
Hepatitis C	ko05160	5.90E-04	8.23E-03
Influenza A	ko05164	9.01E-04	1.02E-02
HTLV-I infection	ko05166	2.58E-03	1.98E-02
Jak-STAT signaling pathway	ko04630	3.84E-03	2.64E-02
Notch signaling pathway	ko04330	4.12E-03	2.75E-02
RIG-I-like receptor signaling pathway	ko04622	5.00E-03	3.27E-02
Viral myocarditis	ko05416	5.16E-03	3.29E-02

### Analysis of targeted genes in primarily immune-related KEGG pathway

To further understand the regulatory roles of miRNAs in immune pathways under pIC challenge, targeted genes in predicted immune-related KEGG pathways were analyzed (Supplementary Table 5). We found that some miRNAs could regulate many typically immune-related targeted genes belonging to these immune pathways by being differentially expressed during the immune response. For example, bbe-miR-4856a-5p regulates the expression of genes encoding cytokine receptor domeless and interleukin 17D (IL-17D) in the Jak-STAT signaling pathway; bbe-miR-210-3p regulates genes encoding Ras-related protein Rab-7A (RAB7A) and NLR family CARD domain-containing protein 4 (NLRC4) in the Salmonella infection pathway; novel_mir49 and novel_mir38 regulate genes encoding TNF receptor-associated factor 4 (TRAF4) and toll-like receptor 1 (TLR1) respectively, in the Hepatitis C pathway; novel_mir49 can regulate TRAF2 and TRAF3, and nuclear factor NF-kappa-B p105 subunit (NFKB1) is a target gene of novel_mir50 in the NF-kappa B signaling pathway; novel_mir196 is a regulator of the gene encoding FAS-associated death domain protein (FADD) in the RIG-I-like receptor signaling pathway, etc. Additionally, some target genes were shared by multiple pathways, such as *TRAF3* and *TRAF4*, and *NFKB1*. Thus a miRNA could regulate multiple target genes and pathways at same time. Moreover, the same target gene was regulated by different miRNAs; for instance, the gene encoding Ras-related protein Rab-7A (RAB7A) was the target of bbe-miR-4856a-5p as well as bbe-miR-210-3p in amphioxus under pIC challenge.

## DISCUSSION

Innate immunity is the first line of defense against exogenous infection in all multicellular organisms. To date, only vertebrates have been demonstrated to possess adaptive immunity [10, 26]. The original adaptive immunity could be traced to an early stage of vertebrate evolution, the jawless vertebrates such as cyclostomes, with more elaborate adaptive immune systems being developed in bony fish [27-29]. Thus, amphioxus must rely on the innate immune system in host defense against pathogen invasion due to the absence of adaptive immunity [4, 10]. To avoid autoimmune damage, the immune responses must be precisely regulated by other non-coding molecules, such as miRNAs [30]. The role of miRNAs in regulation of immune responses has been well investigated in higher chordates, but related studies associated with basal chordates such as amphioxus are limited. Particularly, investigation of amphioxus miRNAs involved in immune responses primarily focus on experiments with bacteria/bacterial mimics challenge [9, 10, 21], but miRNAs related to antiviral immune responses in amphioxus have yet not been characterized or analyzed. Here, we analyzed the genome-wide expression of miRNAs in *B. belcheri* treated by pIC and PBS (negative control) to identify antiviral responsive miRNAs using deep sequencing and speculate their regulatory role for target genes in immune-related pathways.

The results showed that the expression levels of two conserved miRNA sequences from a total of nine conserved miRNA sequences were significantly different in response to pIC challenge (fold-change > 3). These two conserved miRNAs are homologous with bfl-miR-100-3p and bfl-miR-4871-3p. Previous studies have found that down-regulation of miR-100 promoted a decrease in virus genome copies in shrimp and virus-infected shrimp mortality [31]. This inconsistency may be explained by amphioxus-specific (conserved between *B. floridae* and *B. belcheri*) miR-100 and different immune mechanisms between amphioxus and vertebrates. miRNAs homologous to bfl-miR-4871-3p were identified in *B. belcheri* infected with *V. parahemolyticus*, but significant expression of this miRNA was not detected between the treatment and control [10], indicating that amphioxus employs different miRNA molecules to regulate immune responses against virus and bacteria. In addition, analysis for target genes of three novel (belonging to *B. belcheri*-specific miRNAs) miRNAs with the most up-regulation after pIC challenge, predicted three genes (*LOX2*, *RAR*, *GLYR1*) associated with the metabolism of eicosanoids and retinoic acid, demethylation [32] to be the targets of novel_mir157, novel_mir183 and novel_mir121, respectively. Therefore, the highly up-regulated miRNAs in *B. belcheri* challenged by pIC negatively regulated part of genes are involved in metabolism and demethylation, perhaps to ensure adequate energy supply during immune responses by decreasing energy consumption in non-immune related biological processes. Interestingly, *HSP70*, NLR protein family, and *C1qL* are important innate immune molecules in the defense response of host to virus invasion [33, 34]. The most down-regulated molecules, novel-mir138, novel-mir158 and novel-mir140 after pIC challenge promoted expression of key immune-related genes, indicating that these three novel miRNAs may regulate viral stress resistance by directly targeting immune-related genes.

33 known miRNAs with significant differential expression were identified in the treatment groups when compared with the negative control, indicating that these miRNAs participated in innate immune response in *B. belcheri* under pIC challenge. Based on sequence search in the miRBase database, some of these miRNAs were found to be conserved between vertebrates (such as *Homo sapiens*, hsa; *Danio rerio*, dre) and amphioxus, suggesting that they play fundamental roles in the immune system [9]. bbe-miR-92d-3p is an evolutionarily conserved molecule with hsa-miR-92a-3p, dre-miR-92a-3p, etc. Liu et al. found that miR-92a was down-regulated in swine testis cells infected with transmissible gastroenteritis virus (Coronaviridae family) [35]. Besides, using bioinformatics and hybrid PCR methods, Yang et al. detected reduced expression of bbe-miR-92d in *B.belcheri* infected with bacteria, and identified that miR-92d is a regulator for the complement pathway via targeting of C3 [21]. In this study, we found up-regulation of bbe-miR-92d-3p after pIC challenge and the complement component C3 was predicted to be one of its target genes. This indicated that bbe-miR-92d-3p may be a regulator for acute immune response upon virus challenge by targeting C3 in the complement pathway. bbe-miR-10c-5p is well conserved with dre-miR-10c-5p and hsa-miR-10a-5p, etc., and belongs to the miR-10 family, which is highly conserved among different species [36]. Tu et al. demonstrated that miR-10 family plays a key role in suppressing proliferation and inducing apoptosis in humans, mouse and rat ovarian granulosa cells [36]. The up regulation of bbe-miR-10c-5p in our results suggested that apoptosis is an important biological process in antiviral immunity of amphioxus. bbe-miR-252a-5p and bbe-miR-242-5p are well conserved between amphioxus and insects, and deuterostomes (such as *Saccoglossus kowalevskii*, sko, *Strongylocentrotus purpuratus*, spu), suggesting that they are invertebrate-specific. Previous investigation reported that miR-252 suppressed the abundance of dengue virus transcript encoding envelope protein, and then decreased virus replication in *Aedes albopictus* [37]. The function of miR-242 has not been explored so far. Here, we revealed for the first time the immune response of miR-242 to pIC challenge using deep sequencing in amphioxus. Notably, Liao et al. screened 49 significantly up-regulated and seven down-regulated miRNAs in the gill tissue of *B.belcheri* challenged by LPS after 12 post-injection (hpi) [9]. Jin et al. screened 14 significantly differentially expressed miRNAs in the whole body of *B.belcheri* infected with *V. parahemolyticus* [10]. In accordance with our results, 10 up-regulated miRNAs from Liao et al. research results and one (bbe-miR-210) reported by Jin et al. were included in the list of the up-regulated miRNAs in our results, respectively. Two miRNAs (bbe-miR-33 and bbe-miR-92d-3p) screened by Liao et al. and none reported by Jin et al. in down-regulated miRNA were found to overlap with down-regulated miRNAs in this study, respectively. However, we also found that the fold changes in expression compared to the control group were greater or less. This inconsistency may largely be caused by different immunogenic stimuli (bacterial mimics/bacteria versus viral mimics).

The function of miRNAs appears to be that of gene regulation by targeting specific transcripts for inhibition of gene expression. Thus, functional analysis of the potential target mRNAs is essential for revealing miRNA-mediated biological processes and pathways in antiviral immune response of amphioxus. In this study, the set of predicted target genes of DEMs was significantly enriched in different GO and KEGG terms. The GO terms having the most target genes are primarily involved in regulation, cell and single-organisms process. These observations are consistent with the results from immune response analysis of miRNAs to *Cryptocryon irritans* challenge in liver and gill of *L. crocea* [22]. Du et al. performed GO enrichment analysis of differentially expressed genes (DEGs) screened by transcriptome profiling of the spleen of *Schizothorax prenanti* challenged by pIC versus that of control. Three GO terms (related to immune response) obtained in our current study, namely immune system process, response to stimulus, and signaling, were among the 19 GO terms from Du et al. [38]. Huang et al. reported that majority of the immune gene repertoire of amphioxus was well conserved and was shared with vertebrates [39]. Interestingly, despite a high functional overlap of potential target genes of DEMs between amphioxus and bony fish upon immune challenge, conserved DEMs with vertebrates account for a very low percentage (such as 9/197 novel miRNAs, 4.6%). We inferred that the miRNA target site sequence in the 3’ UTR of immune-related genes is more variable than the protein-coding regions, which may confer better evolutionary adaptation to formation of more complex regulatory networks. Certainly, this speculation needs to be further determined systematically by comparative genomic analysis.

At least 14 of the 42 significantly enriched pathways identified in this study were mainly immune-related. The results revealed some of the important immune pathways participating in the immune response of *B. belcheri* to pIC challenge. In addition, some pathways involved in well-known human viral diseases, such as viral carcinogenesis, hepatitis C, influenza A, etc., were mainly detected in the list of immune-related KEGGs. Thus, amphioxus may be a potential model for investigating evolution of signaling pathways associated with human viral diseases. Subsequently, we found that miRNA has regulatory functions in antiviral immune response by targeting key genes involved in innate immune signaling pathways. Many of the target genes were found to encode cytokines such as IL-17D, cytokine receptor domeless (DOME) and interferon-induced helicase C domain-containing protein 1 (IFIH1). Previous studies have demonstrated that negative regulation of cytokines by increasing miRNA enhances interferon-mediated suppression of hepatitis B [40]. A subset of target genes encode pattern recognition receptors (PRRs) expressed by the innate immune cells to identify pathogen molecules, such as those in the TLR and NLR families. Changes in the abundance of these proteins could impact antiviral signaling [41], and hence the predicted regulatory function of miRNA for PRRs in amphioxus under virus invasion. Signal transducers and caspases involved in antiviral signaling, such as TRAF2, 3, 4, Caspase8 (CASP8), were found to be encoded by our predicted target genes [42, 43]. Certainly, except for the above-mentioned, these target genes are involved in the innate immune system, and genes belonging to primarily immune-related pathways were also predicted in this study. These results indicated that known and newly identified DEMs are key regulators for important immune pathways by targeting PRRs, adaptors, cytokines and downstream signal transducers, etc., in antiviral immune response of *B. belcheri*. A part from these, a single miRNA can be a regulator of multiple target genes, while single target genes regulated by multiple miRNAs were also frequently detected here, suggesting the emergence of a complex and diverse miRNA-regulatory network in antiviral immune system in basal chordates.

In summary, deep-sequencing of the treatment and negative control groups was performed on three biological replicates, and the results were further confirmed by qRT-PCR analysis. Moreover, we identified conserved and new miRNAs, and screened transcriptome-wide DEMs against viral mimics (pIC) in *B. belcheri* via deep sequencing and bioinformatics analysis. To explore the regulatory role of the miRNAs identified upon pIC challenge, we predicted and discussed the functions of the target genes of DEMs. Analysis of primarily immune-related target genes, their biological process and signaling pathways involved in antiviral immune response will deepen our understanding of antiviral immune function of post-transcriptional regulation of mRNAs in amphioxus. Additionally, miRNAs with acute immune responses will be preferential candidates for further functional analyses involving antiviral immunity of amphioxus. These results will provide useful information for investigating the miRNA evolution in antiviral immunity in vertebrates in future.

## MATERIALS AND METHODS

### *B.belcheri* samples and viral mimics challenge

Healthy *B. belcheri* adults were obtained from Beihai Marine Station of Nanjing University at Beihai (Guangxi Province, China), and were maintained as described earlier [9, 44]. The amphioxus were acclimatized for about 6 days prior to challenge with pIC to empty the contents of the amphioxus bodies. Approximately 54 adult individuals were equally divided into six groups (9 amphioxiin each group). Next, 15 μl pIC (dissolved in PBS, 100 μg/ml final concentration) was injected into the enterocoelia of each individual in the treatment group, after which they were carefully put back into filtered seawater. An equal dose of PBS was injected in the controls. Six individuals (three pIC treatments and three controls) were collected at each time-point, namely, 2, 6, and 12 hours post-injection (hpi) after pIC challenge. All samples (whole bodies of *B.belcheri*) were immediately frozen in liquid nitrogen for RNA extraction. Three biological replicates were used for each of the treated and negative control groups.

### RNA extraction, library construction and deep sequencing

Total RNA was extracted from the whole body of each adult *B. belcheri* in the control and treatment groups using mirvanaTM miRNA isolation kit AM1560 (Ambion, USA) according to the manufacturer’s protocol. We used Agilent 2100 Bioanalyzer (Agilent Technologies, USA) to detect RNA quality (RIN value) ≥ 7) and estimate the concentration. Equal amount of total RNA from each individual (9 individuals, 3 amphixous at each time-point) from treatment groups was pooled as treatment samples, and likewise from controls as control samples. Pooling of RNA samples at various time points after pIC challenge prior to sequencing may lead to bias in the results (e.g. a miRNA may first be up-regulated and down-regulated subsequently). However, we aimed at obtaining an overview of what occurs during immune challenge rather than understanding the dynamic changes in miRNA expression. Therefore, RNA sampling methods used in this study were similar to that in other experiments [45, 46]. We generated three biological replicates each for the control and treated samples. Preparation of miRNA libraries was then performed according to the standard procedure at Beijing Genomics Institute (BGI, China). 1) RNA segments were separated for size using polypropylene acyl amine gel electrophoresis (PAGE), and 18-30nt longRNA molecules were selected and enriched; 2) 5-adenylated, 3-blocked single-stranded DNA adapters were linked to the 3’-termini of enriched small RNAs; 3) Reverse primer was hybridized to the 3’-termini of RNA; 4) 5’-termini were linked to 5’adaptors; 5) cDNA was synthesized via reverse extension of the RT primer using Superscript II reverse transcriptase (Invitrogen, USA). 6) PCR amplification and cDNA enrichment was performed using both 3’ and 5’adaptors. 7) cDNA libraries were purified by PAGE; 8) Quantitative analysis of the library was performed using Agilent 2100 Bioanalyzer (Agilent Technologies, USA). The prepared cDNA libraries were sequenced on the BGISEQ-500 platform (BGI, China; http://www.seq50.com/en/).

### Preprocessing analysis and identification of *B. belcheri* miRNA

A total of six libraries were sequenced, with three biological replicates of treated and control samples each. The raw tags generated by deep sequencing were filtered by removing low-quality tags, tags with 5’ primer contaminants and poly A stretches, tags without 3’ primer and insertions, and tags of size shorter than 18nt. After filtering, the length distribution of clean tags was analyzed, and clean tags were mapped to the *B. belcheri* genome (ftp://ftp.ncbi.nlm.nih.gov/genomes/, version: Haploidv18h27) using Bowtie2 with the following options: -q -L 16 —phred64 -p 6 [47]. Tags which matched perfectly were retained for downstream analysis. Non-coding small RNAs (sRNA), including rRNAs, snoRNAs, snRNAs, tRNAs, and other sRNA, were annotated by BLAST search in the NCBI GenBank and Rfam (http://rfam.sanger.ac.uk/) databases. After removal of repetitive sequences using RepeatMasker software (http://www.repeatmasker.org/), the remaining sRNAs tags were mapped to mRNA and were subsequently removed to avoid redundancy. The filtered tags were annotated by aligning to the *B. belcheri* miRNA database in miRBase 21.0 (http://www.mirbase.org/index.shtml) to obtain known miRNAs. The remaining non-annotated tags were used to predict novel miRNAs by mirDeep2 (https://www.mdc-berlin.de/8551903/en/) with default options recommended by the software reports [48]. mirDeep2 is a suitable tool that can be used for discovery of miRNAs from deep sequencing data of animal clades, and can identify canonical and non-canonical miRNAs with an accuracy of 98.6-99.9% by a substantially improved algorithm [48, 49]. To obtain a rigorous result, the intersection of identified miRNAs in the three biological replicates was retained as the miRNA set of control or treated groups, and the secondary structures of novel miRNA precursor (pre-miRNAs) were analyzed using RNAfold (http://hackage.haskell.org/package/RNAFold). Furthermore, the predicted novel miRNA tags/sequences with mapped tags in less than 5 were discarded, and the remainder was retained as the final novel miRNA set. The novel miRNAs were annotated by searching in the animal database of miRBase21.0, allowing for a maximum of two mismatches, and those annotated to known miRNAs of other animals were considered to be conserved.

### Identification of differentially expressed miRNA under pIC challenge

The expression levels of all sRNAs in the six libraries were normalized by TPM values: absolute tag count multiply 10^6^/total count of clean tags [22]. We evaluated repeatability among the three biological replicates by pairwise comparison of the samples based on TPM values, and calculated Pearson’s correlations of each pair. Some miRNAs were expressed in only one of control and treated groups, but not in another, indicating that expression of these miRNAs was related to pIC challenge. To avoid the omitting these miRNAs, the union of identified miRNAs in control and treated groups was taken as the final list of miRNAs for analysis. DEMs between the control and treated groups (treatment vs. control) were screened using DEGseq software, which is an R program suitable for different expression analysis of deep sequencing in biological replicates [50]. miRNAs with fold change (FC) ≥ 2 (|log_2_ ratio| ≥ 1) and *P*-values corrected by the false discovery rate (FDR) < 0.001 were considered to be significantly differentially expressed.

### Target gene prediction and functional enrichment analysis of DEMs

To understand the regulatory function of DEMs in the immune response of *B. belcheri* to pIC challenge, the potential target genes of DEMs were predicted using two approaches: RNAhybrid (https://bibiserv.cebitec.uni-bielefeld.de/download/tools/rnahybrid.html), and miRanda (http://www.microrna.org/microrna/getDownloads.do). The intersection of the two prediction results was then taken as the reliable set of target genes of DEMs for further analysis.

To further explore the functions of target genes that were regulated by DEMs in anti-pIC immunity in biological processes and signaling pathways, all protein-coding genes were first annotated against the GO database (http://www.geneontology.org/), non-redundant (NR), UniProt (http://www.uniprot.org/), and KEGG (http://www.genome.jp/kegg) databases. The GO enrichment analysis for the target genes of the DEMs was performed by Fisher’s exact test (*P*-values) in Blast2GO pipeline [51], and *P*-values were used to conduct multiple test correction by FDR. GO terms with FDR< 0*.*05 were considered to be significantly enriched. Moreover, we reduced the redundant terms in the list of significantly enriched GO terms using GO trimming tool (http://lucy.ceh.uvic.ca/go_trimming/cbr_go_trimming.py). KEGG enrichment analysis of target genes of miRNAs was implemented in KOBAS 2.0 software [52], and terms with significantly enriched values (FDR value < 0*.*05) were retained.

### Validation of miRNA deep sequencing data

For validation of the miRNA deep sequencing results, 10 DEMs (five up- and five down-regulated) were randomly selected, and a portion of the RNA preparations used in miRNA deep sequencing was used for qRT-PCR analysis. Total RNA was treated by RNase-free DNase (Qiagen, Germany) to remove any residual DNA contamination. Reverse transcription of total RNA was performed using TransScript Green miRNA Two-Step qRT-PCR SuperMix (TransGenBiotech, China) according to the recommendations in the product manual. We used TransStart TipGreen qPCR SuperMix (TransGen Biotech, China) to conduct qRT-PCR analysis on an ABI 7300 Real-time PCR system (Applied Biosystems, USA) following reaction conditions suggested by TransGenBiotech. All reactions were analyzed in triplicate. The relative quantification of each miRNA was calculated based on the 2^-ΔΔCT^ method [53]. All primers are listed in Supplementary Table 6. A U6 gene was selected as the internal control. IBM SPSS Statistics 22 was employed for statistical analysis.

## SUPPLEMENTARY MATERIALS FIGURE AND TABLES




