# Supplementary material for: Transcriptome-wide analysis of immune-responsive microRNAs against poly (I:C) challenge in Branchiostoma belcheri by deep sequencing and bioinformatics
Source: Oncotarget. 2017 Aug 28;8(43):73590–602. doi: 10.18632/oncotarget.20570 (PMC5650284; doi:10.18632/oncotarget.20570)
Supplement: Supplementary file 1 [file oncotarget-08-73590-s001.pdf]

Transcriptome-wide analysis of immune-responsive microRNAs against poly (I:C) challenge in *Branchiostoma belcheri* by deep sequencing and bioinformatics

SUPPLEMENTARY MATERIALS

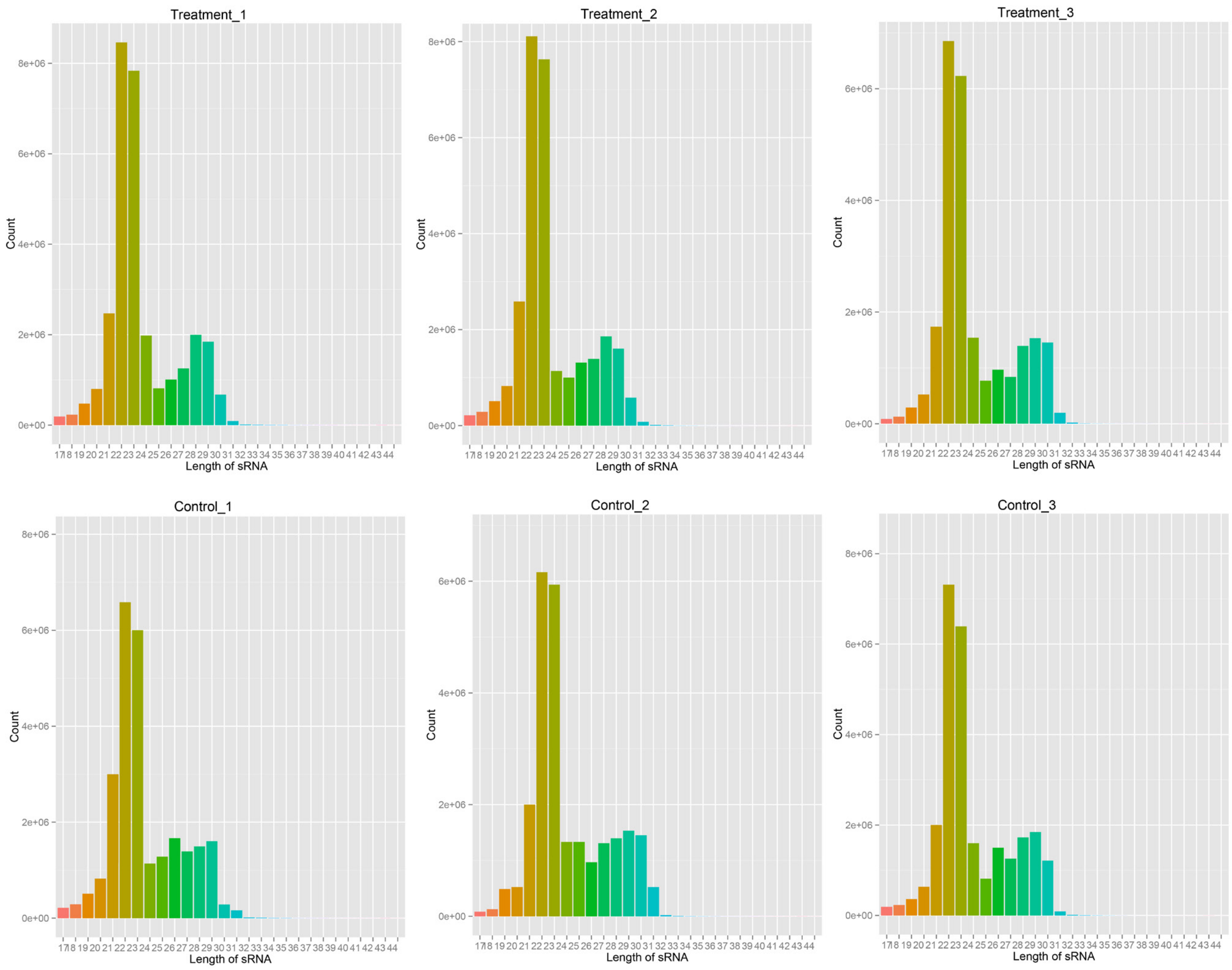

Supplementary Figure 1: Size-class distribution of sRNAs in libraries of control and treatment groups.

Supplementary Table 1: Summary of deep sequencing information for each sample.

Category	Control			Treatment		
	Control_rep1	Control_rep2	Control_rep3	pIC_rep1	pIC_rep2	pIC_rep3
Raw tags	28,903,383	29,591,548	30,135,882	31,297,892	29,856,971	30,077,907
Clean tags (%)	28,217,786 (97.63)	28,742,324 (97.13)	29,235,146 (97.01)	29,965,063 (95.74)	28,815,576 (96.51)	29,191,246 (97.05)
Total clean tags mapping to genome (%)	79.09	79.41	78.82	83.18	83.74	81.47
miRNA tag (%)	8,801,127 (31.19)	8,526,768 (29.67)	8,325,221 (28.48)	11,419,686 (38.11)	11,310,345 (39.25)	11,728,527 (40.18)
Known miRNAs	162	165	164	169	168	168
Novel miRNAs	211	222	215	217	220	214

Supplementary Table 2: Top 30 known miRNA identified in the B. belcheri control and that challenged with pIC.

Id	Family	Average_control_TPM	Average_treatment_TPM
bbe-let-7a-2-3p	let-7	2172.32	185891.61
bbe-let-7a-3p	let-7	1243.52	123348.65
bbe-let-7a-5p	let-7	9485.79	63595.86
bbe-miR-10a-5p	mir-10	847.58	67538.38
bbe-miR-100-3p	mir-100	8736.69	40862.07
bbe-miR-100-5p	mir-100	17634.04	31875.20
bbe-miR-10c-5p	mir-10	55.60	45692.51
bbe-miR-10a-3p	mir-10	772.44	44091.07
bbe-miR-10b-3p	mir-10	678.58	37706.81
bbe-miR-125a-3p	mir-125	311.81	35424.24
bbe-miR-137-3p	mir-137	4.81	29574.46
bbe-miR-2069-3p	mir-2069	9.79	25787.58
bbe-miR-124-5p	mir-124	2.05	25721.47
bbe-miR-2063-3p	mir-2063	0.93	20464.13
bbe-miR-92b-3p	mir-25	18340.31	1.18
bbe-miR-10b-5p	mir-10	474.57	17437.20
bbe-miR-125b-3p	mir-125	95.26	17782.68
bbe-miR-125a-5p	mir-125	282.10	13998.12
bbe-miR-2060a-3p	mir-2060	0.08	11190.28
bbe-miR-129a-3p	mir-129	0.50	9809.73
bbe-miR-135a-3p	mir-135	99.94	8995.85
bbe-miR-133-3p	mir-133	0.16	8537.26
bbe-miR-129b-5p	mir-129	0.00	7175.64
bbe-miR-129a-5p	mir-129	0.59	6472.75
bbe-miR-1-3p	mir-1	0.04	6053.53
bbe-miR-135a-5p	mir-135	398.36	5513.87
bbe-miR-133-5p	mir-133	607.82	5222.60
bbe-miR-92a-3p	mir-92	5488.27	0.00
bbe-miR-2057-3p	mir-2057	427.54	4347.71
bbe-miR-153-3p	mir-153	0.75	4689.24

Supplementary Table 3: Top 30 novel miRNA identified in the B. belcheri control and that challenged with pIC.

Id	Location	Mature sequences (5'- 3')	Expression values in control (TPM)	Expression values in treatment (TPM)
novel_mir51	NW_017804298:1615783:1_1615720	uuuggcacugguacuuuggagu	3278.79	17782.68
novel_mir2	NW_017804043:1856531:1_1856471	acgcguuuaugucugcgccugu	107.45	446.26
novel_mir108	NW_017804464:1170457:1_1170396	gauguuuguacugucugucuguu	91.60	432.37
novel_mir122	NW_017804254:1382901:1_1382827	gguucgauucccggcauggg	54.19	308.07
novel_mir1	NW_017802636:57725:1_57668	cacuggcccucuacgucuuga	112.29	196.17
novel_mir125	NW_017802991:130339:1_130252	uuguccccggcaguugugg	32.36	261.34
novel_mir4	NW_017802636:61269:1_61202	uacuggcucgccaagucuuggu	29.29	181.04
novel_mir5	NW_017803943:382537:1_382478	uaguugccgggcguggaaaagu	23.11	157.68
novel_mir147	NW_017802380:3881206:1_3881160	aguaccgccagagagaccuuu	4.16	165.63
novel_mir3	NW_017804043:1855755:1_1855695	ucgcauugacgucagcgccggc	37.03	98.90
novel_mir6	NW_017802636:54788:1_54728	aacuggcuuucuauguucuugu	34.23	82.69
novel_mir8	NW_017804464:2487832:1_2487773	ugcugaggguuugauucuaugu	10.30	100.78
novel_mir7	NW_017803157:1989589:1_1989530	aaggacuuaaggggacagucgg	28.35	63.39
novel_mir50	NW_017802379:9849414:1_9849356	aggaagacauggucccaguugu	3.10	87.90
novel_mir114	NW_017802781:3374:1_3302	agucaccgucugucggcccguacga	3.06	41.84
novel_mir173	NW_017803919:70437:1_70378	acuggguccagccggcucucccug	4.54	39.83
novel_mir150	NW_017804464:2495455:1_2495396	ucuugggcauguagguuaugag	2.63	40.17
novel_mir11	NW_017802491:3157119:1_3157059	ggaacggagaaugccguaguugu	7.35	31.32
novel_mir14	NW_017803943:384553:1_384495	uagcuggcuggcaaagaaaagu	4.74	30.46
novel_mir16	NW_017804629:4659:1_4601	uguucuucgccacggcaguuuugu	3.34	24.79
novel_mir13	NW_017804120:1405143:1_1405086	cucuugccuggagagccgugcc	5.52	20.33
novel_mir130	NW_017803941:59115:1_59025	caucgacgccguccguuguagacgc	12.27	12.39
novel_mir19	NW_017802379:9931018:1_9930956	auucggguuuauaggaacauga	2.34	21.28
novel_mir18	NW_017802636:58404:1_58345	cgggacuucgcgagucagacgg	7.35	15.36
novel_mir149	NW_017804464:2490346:1_2490288	ugcugaggguuugguucuaugu	2.28	19.91
novel_mir138	NW_017803769:232563:1_232495	gccgagccggggcaggucgccccc	19.09	0.27
novel_mir82	NW_017803158:232597:1_232530	guaccaucuggauuuucgcu	2.13	16.87
novel_mir164	NW_017804464:3138824:1_3138765	uuggguuaucaauagagcuuca	2.48	16.50
novel_mir181	NW_017804265:514561:1_514494	uguucgaaucacgucggggu	3.12	15.70
novel_mir69	NW_017802414:43994:1_43928	uugcguagcgaaaauccggaug	3.89	14.25

Supplementary Table 4: List of the KEGG pathway of targeted genes of differentially expressed miRNA between B. belcheri control and that challenged with pIC.

Pathway	Pathway id	P-value	FDR
Tight junction	ko04530	3.16E-08	9.71E-05
Dilated cardiomyopathy	ko05414	3.50E-07	5.37E-05
Arrhythmogenic right ventricular cardiomyopathy (ARVC)	ko05412	1.25E-06	1.28E-04
Platelet activation	ko04611	4.61E-06	3.54E-04
Retrograde endocannabinoid signaling	ko04723	6.12E-06	3.76E-04
Adherens junction	ko04520	8.73E-06	4.46E-04
Carbohydrate digestion and absorption	ko04973	1.30E-05	5.70E-04
Hypertrophic cardiomyopathy (HCM)	ko05410	5.57E-05	2.14E-03
NF-kappa B signaling pathway	ko04064	7.70E-05	2.63E-03
NOD-like receptor signaling pathway	ko04621	8.64E-05	2.65E-03
MAPK signaling pathway	ko04010	1.10E-04	2.90E-03
Thyroid hormone signaling pathway	ko04919	1.20E-04	2.90E-03
Type II diabetes mellitus	ko04930	1.23E-04	2.90E-03
Endocytosis	ko04144	1.38E-04	3.03E-03
GABAergic synapse	ko04727	1.63E-04	3.35E-03
Chemokine signaling pathway	ko04062	1.97E-04	3.78E-03
Viral carcinogenesis	ko05203	2.11E-04	3.81E-03
Cholinergic synapse	ko04725	2.97E-04	5.07E-03
Salmonella infection	ko05132	3.77E-04	6.09E-03
Galactose metabolism	ko00052	5.15E-04	7.73E-03
RNA transport	ko03013	5.29E-04	7.73E-03
Hepatitis C	ko05160	5.90E-04	8.23E-03
Ubiquitin mediated proteolysis	ko04120	6.62E-04	8.84E-03
Focal adhesion	ko04510	7.77E-04	9.71E-03
Cardiac muscle contraction	ko04260	7.91E-04	9.71E-03
MicroRNAs in cancer	ko05206	8.41E-04	9.93E-03
Influenza A	ko05164	9.01E-04	1.02E-02
Starch and sucrose metabolism	ko00500	1.07E-03	1.17E-02
ECM-receptor interaction	ko04512	1.20E-03	1.27E-02
ErbB signaling pathway	ko04012	1.35E-03	1.34E-02
Progesterone-mediated oocyte maturation	ko04914	1.35E-03	1.34E-02
Central carbon metabolism in cancer	ko05230	1.67E-03	1.59E-02
Glycolysis / Gluconeogenesis	ko00010	1.71E-03	1.59E-02
HTLV-I infection	ko05166	2.58E-03	1.98E-02
Vasopressin-regulated water reabsorption	ko04962	2.82E-03	2.10E-02
Serotonergic synapse	ko04726	2.87E-03	2.10E-02
Prion diseases	ko05020	3.63E-03	2.59E-02
Jak-STAT signaling pathway	ko04630	3.84E-03	2.64E-02
Pancreatic secretion	ko04972	3.87E-03	2.64E-02
Notch signaling pathway	ko04330	4.12E-03	2.75E-02
RIG-I-like receptor signaling pathway	ko04622	5.00E-03	3.27E-02
Viral myocarditis	ko05416	5.16E-03	3.29E-02

Supplementary Table 5: The predicted immune-related KEGG pathway analysis of targeted genes in B. belcheri challenged by poly (I:C).

Supplementary Table 6: Primers used in quantitative RT-PCR.

Gene id	Primer sequence (5'-3')
bbe-miR-184-5p	GGCGGCCCTTATCACTTCTTCC
bbe-miR-200b-3p	TTCATCGGCTTACCAGGCAGCA
bbe-miR-4887-5p	TGCGGCGGTTCTCGATTGTTCT
bbe-miR-2070-5p	TGGAGGTGGTTTCCACAGCCTCTAC
bbe-miR-4869-3p	GTTGCGGAGGGACCTGTAGTCAAC
bbe-miR-92d-3p	GGGCGGCTATTGCACTTATCCTG
novel_mir114	ATAAAGTCACCGTCTGTCGGCCC
novel_mir193	GCGCAATCCACAGTGATTTTGG
novel_mir130	ATCATCGACGCCGTCCGTTGT
novel_mir148	GCGTTGGGAGTCAGGACACCAT
