# Supplementary material for: Transcriptome-wide analysis of immune-responsive microRNAs against poly (I:C) challenge in Branchiostoma belcheri by deep sequencing and bioinformatics
Source: Oncotarget. 2017 Aug 28;8(43):73590–602. doi: 10.18632/oncotarget.20570 (PMC5650284; doi:10.18632/oncotarget.20570)
Supplement: Supplementary file 2 [file oncotarget-08-73590-s002.docx]

**Supplementary Table 5: The predicted immune-related KEGG pathway analysis of targeted genes in *B. belcheri* challenged by poly (I:C).**

| **Pathway (ID)** | **miRNA** | **Fold change** | **Target gene description** |
| --- | --- | --- | --- |
| Jak-STAT signaling pathway (ko04630) | bbe-miR-210-3p | 2.24 | E3 SUMO-protein ligase PIAS2 |
|  |  |  | cytokine receptor domeless |
|  |  |  | internexin neuronal intermediate filament protein, alpha |
|  |  |  | E3 SUMO-protein ligase PIAS2 |
|  | novel_mir170 | 1.51 | E3 SUMO-protein ligase PIAS2 |
|  | novel_mir10 | 1.58 | B-Raf proto-oncogene serine/threonine-protein kinase |
|  |  |  | interleukin 17D |
|  | bbe-miR-33-3p | -2.16 | Myc proto-oncogene protein |
|  |  |  | E1A/CREB-binding protein |
|  | bbe-miR-4856a-5p | 3.48 | Myc proto-oncogene protein |
|  |  |  | internexin neuronal intermediate filament protein, alpha |
|  |  |  | cytokine receptor domeless |
|  |  |  | interleukin 17D |
|  | novel_mir130 | -2.12 | ADP-ribosylation factor-like protein 9 |
|  | novel_mir148 | -3.51 | suppressor of cytokine signaling 6/7 |
|  |  |  | E1A/CREB-binding protein |
|  | bbe-miR-10c-5p | 2.52 | signal transducer and activator of transcription 5B |
|  | novel_mir166 | -1.09 | son of sevenless |
|  | novel_mir26 | 1.16 | E1A/CREB-binding protein |
| Salmonella infection (ko05132) | novel_mir150 | 1.79 | NLR family CARD domain-containing protein 4 |
|  | bbe-miR-184-5p | 2.73 | golgin subfamily A member 4 |
|  | bbe-miR-7-3p | -1.17 | nuclear receptor co-repressor 1 |
|  | novel_mir31 | 1.47 | NLR family CARD domain-containing protein 4 |
|  | novel_mir85 | 5.28 | golgin subfamily A member 4 |
|  |  |  | NLR family CARD domain-containing protein 4 |
|  |  |  | actin beta/gamma 1 |
|  | novel_mir166 | -1.09 | tight junction protein 1 |
|  |  |  | actin beta/gamma 1 |
|  | bbe-miR-4856a-5p | 3.48 | myosin heavy chain |
|  |  |  | Ras-related protein Rab-7A |
|  | novel_mir147 | 3.16 | actin beta/gamma 1 |
|  | novel_mir65 | 3.20 | mitogen-activated protein kinase 15 |
|  |  |  | golgin subfamily A member 4 |
|  |  |  | NLR family CARD domain-containing protein 4 |
|  | bbe-miR-2071-3p | -2.21 | kinesin light chain |
|  |  |  | centrosomal protein CEP250 |
|  |  |  | myosin heavy chain |
|  | novel_mir95 | 6.04 | NLR family CARD domain-containing protein 4 |
|  | bbe-miR-210-3p | 2.24 | Ras-related protein Rab-7A |
|  |  |  | NLR family CARD domain-containing protein 4 |
| NOD-like receptor signaling pathway (ko04621) | novel_mir193 | 7.25 | nucleotide-binding oligomerization domain-containing protein 1 |
|  |  |  | TAK1-binding protein 1 |
|  | novel_mir150 | 1.79 | erbb2-interacting protein |
|  | bbe-miR-200b-3p | 1.92 | nuclear receptor co-repressor 1 |
|  | novel_mir31 | 1.47 | nucleotide-binding oligomerization domain-containing protein 1 |
|  | novel_mir131 | -3.80 | ubiquitin-like-conjugating enzyme ATG3 |
|  | bbe-miR-4856a-5p | 3.48 | caspase 8 |
| Endocytosis (ko04144) | novel_mir150 | 1.79 | cytochrome c oxidase subunit 7a |
|  |  |  | charged multivesicular body protein 7 |
|  |  |  | nuclear mitotic apparatus protein 1 |
|  | bbe-miR-184-5p | 2.73 | leucine-rich repeats and death domain-containing protein |
|  | bbe-miR-2062-3p | -2.98 | sorting nexin-1/2 |
|  |  |  | insulysin |
| Viral myocarditis (ko05416) | novel_mir150 | 1.79 | myosin heavy chain 6/7 |
|  | bbe-miR-252a-5p | -4.64 | dystrophin |
|  | bbe-miR-34b-3p | 2.71 | laminin, alpha 1/2 |
| Chemokine signaling pathway (ko04062) | novel_mir81 | 3.81 | T-lymphoma invasion and metastasis-inducing protein 1 |
|  |  |  | engulfment and cell motility protein 1 |
|  | novel_mir31 | 1.47 | proto-oncogene C-crk |
|  | bbe-miR-4856a-5p | 3.48 | lipoyltransferase 1 |
|  | novel_mir160 | 3.13 | focal adhesion kinase 1 |
|  |  |  | breast cancer anti-estrogen resistance 1 |
|  | bbe-miR-7-3p | -1.17 | signal transducer and activator of transcription 5B |
|  | novel_mir26 | 1.16 | tyrosine-protein kinase Src |
|  | bbe-miR-33-3p | -2.16 | guanine nucleotide exchange factor VAV |
| Notch signaling pathway (ko04330) | novel_mir130 | -2.12 | ectonucleotide pyrophosphatase/phosphodiesterase family member 7 |
|  |  |  | Notch |
|  | bbe-miR-34b-3p | 2.71 | laminin, alpha 1/2 |
|  | bbe-miR-33-2-3p | 2.76 | hairy and enhancer of split 1 |
|  | bbe-miR-182b-5p | -3.65 | Notch |
| Hepatitis C (ko05160) | novel_mir49 | 3.76 | TNF receptor-associated factor 4 |
|  | novel_mir150 | 1.79 | receptor-interacting serine/threonine-protein kinase 1 |
|  | novel_mir81 | 3.81 | serine/threonine-protein phosphatase 2A regulatory subunit A |
|  | novel_mir38 | 3.39 | toll-like receptor 1 |
|  | bbe-miR-4856a-5p | 3.48 | retinoid X receptor alpha |
|  |  |  | lipoyltransferase 1 |
|  |  |  | 2'-5'-oligoadenylate synthetase |
| HTLV-I infection (ko05166) | novel_mir130 | -2.12 | Myc proto-oncogene protein |
|  |  |  | nuclear factor NF-kappa-B p105 subunit |
|  | novel_mir136 | 4.13 | anaphase-promoting complex subunit 7 |
|  |  |  | Fas-binding factor 1 |
|  | novel_mir156 | 4.23 | adenylate cyclase 2 |
|  |  |  | tumor necrosis factor receptor superfamily member 5 |
|  |  |  | cyclin B |
|  | bbe-miR-4888-5p | -1.23 | anaphase-promoting complex subunit 2 |
|  | novel_mir114 | 1.63 | cytochrome P450, family 2 |
| Influenza A (ko05164) | novel_mir49 | 3.76 | tissue plasminogen activator |
|  | bbe-miR-252a-3p | 1.36 | interferon-induced helicase C domain-containing protein 1 |
|  | novel_mir136 | 4.13 | proprotein convertase subtilisin/kexin type 1 |
|  |  |  | double-stranded RNA-specific adenosine deaminase |
|  | novel_mir81 | 3.81 | DnaJ homolog subfamily C member 3 |
|  |  |  | transmembrane protease serine 9 |
|  |  |  | class II, major histocompatibility complex, transactivator |
|  | novel_mir31 | 1.47 | mitogen-activated protein kinase kinase 6 |
|  |  |  | S-(hydroxymethyl)glutathione dehydrogenase / alcohol dehydrogenase |
| NF-kappa B signaling pathway (ko04064) | novel_mir196 | 7.25 | B-cell linker protein |
|  |  |  | mitochondrial FAD-linked sulfhydryl oxidase |
|  | novel_mir49 | 3.76 | TNF receptor-associated factor 3 |
|  |  |  | TNF receptor-associated factor 2 |
|  | novel_mir150 | 1.79 | receptor-interacting serine/threonine-protein kinase 1 |
|  | bbe-miR-184-5p | 2.73 | cytohesin |
|  | novel_mir50 | 2.67 | nuclear factor NF-kappa-B p105 subunit |
|  | novel_mir1 | -1.34 | apoptosis regulator BCL-W |
| MAPK signaling pathway - fly (ko04013) | novel_mir136 | 4.13 | Ras GTPase-activating protein 3 |
|  | novel_mir33 | 6.89 | GTPase HRas |
|  | novel_mir95 | 6.04 | epidermal growth factor receptor |
|  | novel_mir148 | -3.51 | abelson tyrosine-protein kinase 2 |
|  | bbe-miR-31-3p | 3.66 | ETS translocation variant 6/7 |
| RIG-I-like receptor signaling pathway (ko04622) | novel_mir196 | 7.25 | FAS-associated death domain protein |
|  | novel_mir49 | 3.76 | TNF receptor-associated factor 4 |
|  | novel_mir150 | 1.79 | receptor-interacting serine/threonine-protein kinase 1 |
|  | bbe-miR-252a-3p | 1.36 | interferon-induced helicase C domain-containing protein 1 |
|  | novel_mir50 | 2.67 | nuclear factor NF-kappa-B p105 subunit |
|  | novel_mir136 | 4.13 | TNF receptor-associated factor 3 |
|  | novel_mir138 | -8.30 | triose/dihydroxyacetone kinase / FAD-AMP lyase (cyclizing) |
| Viral carcinogenesis (ko05203) | novel_mir196 | 7.25 | ubiquitin carboxyl-terminal hydrolase 7 |
|  |  |  | echinoderm microtubule-associated protein-like 1/2 |
|  |  |  | histone H2B |
|  | novel_mir49 | 3.76 | TNF receptor-associated factor 4 |
|  | novel_mir50 | 2.67 | nuclear factor NF-kappa-B p105 subunit |
|  | novel_mir166 | -1.09 | pyruvate kinase |
|  | novel_mir31 | 1.47 | vacuole morphology and inheritance protein 14 |
|  |  |  | interleukin 6 signal transducer |
|  | novel_mir35 | 5.56 | G protein-coupled receptor |
|  |  |  | E1A/CREB-binding protein |
|  | novel_mir156 | 4.23 | cyclic AMP-responsive element-binding protein 3 |
|  | bbe-miR-4866-3p | -2.04 | ubiquitin carboxyl-terminal hydrolase 7 |
|  |  |  | SNW domain-containing protein 1 |
|  | bbe-let-7a-2-3p | 1.45 | recombining binding protein suppressor of hairless |
|  |  |  | leucine-rich repeat kinase 2 |
